# Genome-wide profiling of GRAS genes in flax (*Linum usitatissimum* L.) reveals *LuGRAS30* as a key regulator of drought stress resistance

**DOI:** 10.1080/21645698.2025.2548639

**Published:** 2025-08-25

**Authors:** Yihang Bao, Chulin Pan

**Affiliations:** a Department of Plant Sciences, Jilin University, Changchun, China; b College of Biology and Agricultural Engineering, Jilin University, Changchun, China

**Keywords:** Drought stress, GRAS genes, phylogeny analysis

## Abstract

GRAS genes are indispensable for modulating plant growth, developmental patterning, and adaptive responses to biotic and abiotic stress conditions. In this study, 99 *LuGRAS* genes were identified in the flax genome. Phylogenetic analysis classified them into 10 subfamilies: HAM, DELLA, DLT, SCL3, LAS, SCL4/7, SCR, SCL, SHR, and PAT1. Gene structure and motif analyses revealed that *LuGRAS* genes within the same clade exhibited conserved exon-intron organization and motif architectures. Promoter analysis showed that most *LuGRAS* genes contained cis-regulatory elements responsive to plant hormones (MeJA and abscisic acid) and abiotic stresses, including anaerobic induction, low temperature, and drought inducibility. MiRNA target prediction indicated that lus-miR395 is the primary regulatory miRNA for the *LuGRAS* gene family. Expression pattern analysis demonstrated that all *LuGRAS* family members were highly expressed in leaves and roots. qRT-PCR analysis further revealed that 10 genes were significantly upregulated under abiotic stresses (cold, drought, and salt), suggesting their involvement in antioxidant defense mechanisms. In *Arabidopsis*, *LuGRAS30* enhanced drought tolerance by scavenging reactive oxygen species (ROS) accumulation. Subcellular localization analysis demonstrated that *LuGRAS30* was localized in the nucleus. This study provides new insights into the role of *LuGRAS* genes in flax stress tolerance and contributes to flax breeding and further functional research.

## 1. Introduction

The GRAS transcription factors constitute an indispensable class of proteins governing plant growth and developmental processes, and represent one of the earliest discovered and most extensively characterized transcription factor families. The nomenclature “GRAS” derives from the three founding members initially identified within this family: REPRESSOR OF GAI (RGA), GIBBERELLIC-ACID INSENSITIVE (GAI), and SCARECROW (SCR).^1,2^ The C-terminal region of GRAS proteins contains a highly conserved segment universally designated as the GRAS domain. A canonical GRAS protein, typically spanning 400–700 amino acids in length, is structurally organized into five signature conserved motifs: the leucine-rich region I (LHRI), VHIID domain, leucine-rich region II (LHRII), PFYRE motif, and SAW domain.^3,4^ Based on variations in N-terminal length and sequence composition, members of the GRAS gene family can be phylogenetically categorized into up to 17 subfamilies, including DELLA, HAM, PAT1, LISCL, SCR, SHR, SCL3, LAS, and in other plant species: NSP1, SCLB, NSP2, RAD1, SCLA, RAM1, DL, SCL4/7, and SCL32.^5^

GRAS genes influence plant morphogenesis by regulating pathways such as meristem formation, root and shoot development, and light signaling responses. In *Arabidopsis*, the SCR-SHR module establishes the radial structure of roots by controlling the asymmetric division of cortex/endodermis initial cells (CEIs). The SCR protein interacts with the SHR protein, activating the cell cycle gene *CYCLIND6* to promote root cell layer stratification.^6^ The *scr* mutant exhibits defective monocortical layer organization due to the absence of endodermal differentiation.^7^ *OsMOC1* and its ortholog *LAS* function as phylogenetically conserved core regulators of rice tillering morphogenesis, wherein their conserved GRAS domains mediate transcriptional networks essential for lateral meristem initiation and maintenance.^8^ SHR transcriptionally activates the SCR promoter in a tissue-specific manner, critically regulating radial patterning during root and shoot morphogenesis.^9^ The ZmGRAS11 gene lacking the DELLA domain promotes starchy endosperm cell expansion.^10^ In leguminous plants, *NSP1* and *NSP2* form a heterodimer via their conserved GRAS domains, directly binding to light-responsive elements in the promoter regions of phyA downstream target genes, thereby cooperatively activating the phytochrome signaling pathway.^11^ In *Arabidopsis thaliana*, the GRAS family members *RGL1* and *RGL2* regulate plant growth and development through distinct signaling pathways: *RGL1* governs gibberellin (GA) responsiveness via DELLA-dependent repression, while *RGL2* modulates photomorphogenesis by integrating light signaling cascades.^12^

Additionally, GRAS proteins play important roles in both biotic stress and abiotic stress. Classified within the PAT1 subfamily, *OsCIGR1* and *OsCIGR2* function as suppressors of rice pathogen invasion by mediating innate immunity pathways.^13^ Ectopic expression of the wild grapevine *VaPAT1* gene in transgenic *Arabidopsis* markedly improves resistance to abiotic stressors, as evidenced by enhanced survival rates under stress conditions.^14^ Similarly, constitutive expression of *ZmGRAS15* in maize substantially increases drought resilience during early growth stages, primarily through modulation of primary root elongation at the seedling phase.^15^ *GmGRAS37* expression is upregulated in response to drought, salt stress, and abscisic acid (ABA) treatment. Transgenic soybean plants overexpressing *GmGRAS37* exhibit significantly improved tolerance to drought and salt stress.^16^ The GRAS protein *OsDLA* in rice regulates the diterpenoid phytoalexin biosynthesis pathway and synergistically interacts with *GSK2* and *OsWRKY53* to form a functional defense module, thereby enhancing resistance against rice blast.^17^ *GhSCL13-2A*, a *PAT1* subfamily protein in upland cotton, modulates jasmonic acid (JA) and salicylic acid (SA) signaling cascades, thereby amplifying reactive oxygen species (ROS) production and conferring robust resistance to Verticillium wilt.^18^ The overexpression of *BpGRAS34* has been shown to enhance salt tolerance in birch by promoting reactive oxygen species (ROS) scavenging and proline accumulation.^19^

Flax, a historically significant crop with global cultivation spanning temperate zones, is functionally classified into three agronomically distinct types: oil-flax (linseed), fiber-flax, and dual-purpose varieties, reflecting its versatile applications in agriculture and industry.^20,21^ The seeds of flax are rich in bioactive compounds such as lignans, dietary fiber, and alpha-linolenic acid (ALA) – an omega-3 polyunsaturated fatty acid critical for cardiovascular and neurological health in humans.^22^ So far, no comprehensive studies have been conducted on the GRAS gene family in flax. This research systematically examined flax GRAS genes through bioinformatics approaches, exploring their phylogenetic evolution, chromosomal distribution, physicochemical characteristics, sequence motifs, evolutionary relationships, promoter cis-regulatory elements, subcellular localization, and expression patterns, along with their responses to low-temperature, salt, and drought stresses. Furthermore, we explored the molecular mechanism of *LuGRAS30* under drought conditions. These findings provide a foundation for further functional characterization of GRAS genes in flax. This study represents the first comprehensive report on *GRAS* genes in flax.

## 2. Materials and Methods

### 2.1. Plant Material

The experimental procedures were performed using the flax cultivar Longya10. Seed sterilization involved sequential treatments: initial immersion in 75% ethanol for 10 minutes for surface disinfection, subsequent washing with autoclaved deionized water to remove residual ethanol, and final transplantation into pre-sterilized nutrient-rich soil under controlled growth conditions. The temperature and lighting duration of the incubator were set to 26/18°C and 16/8 h. Stress treatments were carried out when flax seedlings grew to 6–7 cm. The drought and salt treatment groups extracted the plants from the soil, thoroughly rinsed them with clean water, and placed them into conical flasks containing 10% polyethylene glycol and 100 mM sodium chloride solutions, respectively. Control plants were maintained in sterile distilled water. For cold stress induction, flax seedlings were transferred to a climate-controlled growth chamber at 4°C. Leaf samples from both control and treated groups were collected simultaneously at 0, 3, 6, and 9 hours post-treatment initiation, with triplicate biological replicates per time point to minimize temporal variability. All harvested tissues were immediately immersed in liquid nitrogen for rapid cryopreservation and subsequently archived at −80°C.

### 2.2. Identification of GRAS Gene in Flax

The complete genome assembly of flax was retrieved from the NCBI database under accession number QMEI02000000. Genome annotation files were sourced from the Figshare repository (https://figshare.com/articles/dataset/Annotation_files_for_ Longya-10_genome/13614311). A cohort of 33 *Arabidopsis* GRAS protein sequences was acquired from TAIR (https://www.arabidopsis.org/).^23^ The conserved GRAS domain profile (PF03514) was extracted from the Pfam database (http://pfam.xfam.org/) using Hidden Markov Model (HMM) analysis.^24^ Flax GRAS genes were also predicted by hmmsearch program of HMMER3.0 software,^25^ followed by stringent filtering and structural validation via SMART (http://smart.embl.de/smart/batch.pl.), yielding a final set of 99 non-redundant *LuGRAS* genes. To systematically characterize these genes, biophysical parameters – including coding sequence length, amino acid composition, molecular weight (MW), theoretical isoelectric point (pI), and grand average of hydropathy (GRAVY) – were computationally derived using the ExPASy ProtParam tool (https://web.expasy.org/protparam/).^26^ The BUSCA net station was used to carry out the sub-cellular location pretest (http://www.busca.cn).

### 2.3. Phylogeny, Chromosome Location, Conserved Domain and Conserved Motif of LuGRAS Gene

Multiple sequence alignment of GRAS protein sequences from *Arabidopsis*, rice, and flax was performed using ClustalW (v11.0) embedded in the MEGA software suite with standard alignment parameters. Subsequently, a maximum likelihood (ML)-based phylogenetic tree was generated through the MEGA platform (v11.0), employing a neighbor-joining algorithm with preset configurations, including 1,000 bootstrap replicates to assess node reliability.^27^ Chromosomal localization of *LuGRAS* genes was determined through alignment of flax genomic FASTA sequences with structural annotation files in GFF3 format. Conserved protein motif composition analysis was conducted via the MEME Suite (http://alternate.meme-suite.org/tools/meme), followed by graphical representation of motif architectures using TBtools v2.069.^28^

### 2.4. Genome-Wide Replication and Collinear Analysis of LuGRAS Gene

Chromosomal assemblies and functional annotation resources for model species such as *Arabidopsis*, maize, and rice were obtained from the Phytozome platform (https://phytozome-next.jgi.doe.gov/). Multiple collinear scanning (MCScanX) toolkits are used to predict collinear relationships.^29^ The repetitive *LuGRAS* gene was identified as genome-wide repeat (WGDs). Tandem repeat genes are two or more homologous genes on chromosomes, the distance is not more than 100kb, and there are no other genes. The fragment repeat gene (Score < 1e-5) was detected by nucleotide BLAST (BLASTN), which contained a range of 100 kb (upstream and downstream 50kb) around the coding sequence (CDS). Gene duplication identification thresholds were defined as aligned genomic segments ≥ 200 base pairs (bp) in length with nucleotide sequence identity exceeding 85%.^30^

### 2.5. miRNA Prediction and Cis-Acting Element Analysis

Putative miRNA binding loci within the 5′/3′ untranslated regions (UTRs) and protein-coding sequences (CDS) of *LuGRAS* genes were computationally predicted utilizing the psRNATarget platform (Plant MicroRNA Target Analysis Server; https://www.zhaolab.org/psRNATarget/analysis?function=3), employing default configurations for sequence alignment.^31^ Promoter regions spanning 2,000 base pairs upstream of all *LuGRAS* genes were isolated using TBtools software. To delineate stress-responsive regulatory mechanisms, cis-acting elements within these promoter sequences were systematically annotated via the PlantCARE database (http://bioinformatics.psb.ugent.be/webtools/plantcare/html/), followed by graphical mapping of the identified motifs using TBtools.

### 2.6. Analysis of Expression Pattern of LuGRAS Gene Family

In this study, transcriptome sequencing data from five flax datasets were used (https://www.ncbi.nlm.nih.gov/sra/term=.): (a) pistil, stamen, fruit, and shoot apex tissues (PRJNA1002756); (b) floral tissues at 30, 20, 10, and 5 days after flowering (PRJNA833557); (c) different flax embryo tissues, anthers, and seed tissues (PRJNA663265); (d) root and leaf tissues under salt stress (PRJNA977728); and (e) stem tissues under heat stress (PRJNA874329). The data were filtered using fastp,^32^ aligned to the Longya10 reference genome, and expression levels were quantified using R. Finally, a heatmap of FPKM log2 values was generated.

### 2.7. RNA Extraction and Fluorescence Quantitative PCR Analysis

The total RNA of flax leaves was extracted by Trizol method. Using M-MLV reverse transcription kit, the first cDNA was generated from total RNA. Oligo 7 was used to design upstream and downstream primers (Table S4), and TB Green ®Pemix ExTaqTMII (Takara Bio, Kyoto, Japan) was used for RT-PCR test. The experimental results were statistically analyzed by 2^−ΔΔCT^ method.^33^ Using GAPDH as the internal control gene, each sample was repeated 3 times to calculate the CT value.

### 2.8. Subcellular Localization and Construction of Transgenic Arabidopsis with LuGRAS30 Gene

The full-length coding sequence of *LuGRAS30* was amplified by PCR (Table S3), cloned into the pGM-T vector, and transformed into *E. coli*. The target fragment was excised from pGM-T-LuGRAS30 via XhoI/SalI digestion and ligated into the similarly digested pGD-GFP vector to generate pGD-*LuGRAS30*-GFP. Both the empty pGD-GFP vector and recombinant plasmid were separately introduced into *Agrobacterium tumefaciens GV3101*. The bacterial culture was resuspended in an infiltration buffer, and after 3 hours of induction at 28°C, the culture was injected into *Nicotiana benthamiana* leaves. After 3 days of dark incubation, GFP fluorescence signals were observed using confocal microscopy.

In this study, the inflorescence soaking method was used to transform *Arabidopsis*. *Agrobacterium* containing *LuGRAS30* was inoculated into LB medium and cultured at 28°C with shaking until OD600 reached 1.0. After centrifugation at 5000 rpm for 10 minutes, the bacterial pellet was resuspended in a solution of 5% sucrose and 0.01% Silwet-77. The inflorescences were submerged in the bacterial suspension for 2 minutes, and the process was repeated three times weekly. After harvesting T1 seeds, positive T2 lines were selected through hygromycin resistance screening. The T3 generation plants exhibiting 100% resistance were identified as homozygous lines.

### 2.9. Detection of Physiological and Biochemical Indicators and Expression analysis of Key Drought-Responsive Genes in Transgenic Arabidopsis Overexpressing LuGRAS30

Uniformly developed wild-type and *LuGRAS30*-overexpressing *Arabidopsis* lines (OE-1, OE-4, OE-5) were transplanted into sterile soil and allocated into two cohorts: well-watered controls and drought-stressed experimental groups. Following a three-week acclimatization period under standard growth conditions, control plants were cultivated under normal irrigation, while the experimental group was subjected to a 21-day natural drought regime. The physiological stress markers, including chlorophyll content, malondialdehyde (MDA) levels, proline (Pro) concentration, the enzymatic activities of superoxide dismutase (SOD) and peroxidase (POD), and hydrogen peroxide (H₂O₂) content, were quantified using commercial assay kits following the manufacturers’ protocols.^34^

Total RNA was extracted from the leaf tissues of *Arabidopsis* under drought stress treatment and control conditions using TRIzol reagent (Invitrogen), and first-strand cDNA was synthesized using a reverse transcription kit (Takara). Quantitative real-time PCR was performed using SYBR Green Master Mix (Roche) on a LightCycler® 480 system. The expression levels of key drought-responsive genes, including *RD22*, *RD29A*, *NCED3*, *ABI1*, *ABI2*, *ABI3*, and *ABI4*, were analyzed. The Actin2 gene was used as an internal control. Relative expression levels were calculated using the 2^−ΔΔCT^ method. Three biological replicates and three technical replicates were conducted for each sample.

## 3. Results

### 3.1. Identification and Chromosome Localization of GRAS Gene Family in Flax

A Hidden Markov Model (HMM) search was employed using the GRAS domain profile (PF03514) from the Pfam database, which screened 99 putative GRAS-encoding genes from the flax genome (Longya10). These loci were systematically assigned sequential identifiers (*LuGRAS1* to *LuGRAS99*) based on their linear arrangement along chromosomes (Table 1). Characterization of biophysical and biochemical parameters revealed substantial variation in *LuGRAS* protein sizes, with polypeptide lengths spanning 319 residues (*LuGRAS7*) to 785 residues (*LuGRAS73*) and molecular masses ranging between 36.11 kDa and 88.00 kDa. The isoelectric point (pI) analysis showed that only 12% (7/58) of the proteins had a pI greater than 7, indicating that the majority of family members tend to be acidic or neutral. In terms of stability, 96.6% of the *LuGRAS* proteins were classified as unstable (Instability Index > 40), while only 3.4% met the stability standard (Instability Index < 40). The aliphatic index varied significantly (63.35–99.68), with 30% of the members having an index above 90, suggesting substantial differences in thermal stability. Additionally, the GRAVY values of the proteins ranged from −0.715 to 0, confirming the strong hydrophilicity of the family, except for *LuGRAS50*, which had a GRAVY value of 0, indicating near-neutral hydrophilicity. Overall, the *LuGRAS* gene family is characterized by moderate molecular weight, a tendency toward neutral or acidic pI, high instability, and significant variation in thermal stability, reflecting its functional diversity. Predictions of subcellular localization suggested that every *LuGRAS* gene was localized in the nucleus.

**Table 1.** Prediction and characterization of GRAS gene in flax.

Gene	Gene ID in Genome	Number of Amino Acids	Molecular Weight (KDa)	PI	Instability Index	Aliphatic Index	Grand Average of Hydropathicity (GRAVY)	Subcellular Localization
LuGRAS1	L.us.o.m.scaffold37.42	496	55.23	7.11	37.98	91.23	−0.249	Nuclear
LuGRAS2	L.us.o.m.scaffold37.7	529	59.17	6.33	50.05	84.39	−0.245	Nuclear
LuGRAS3	L.us.o.m.scaffold37.6	527	59.09	6.62	51.81	80.11	−0.278	Nuclear
LuGRAS4	L.us.o.m.scaffold210.18	517	58.18	5.87	58.56	66.21	−0.48	Nuclear
LuGRAS5	L.us.o.m.scaffold45.296	448	50.17	7.11	49.02	89.04	−0.193	Nuclear
LuGRAS6	L.us.o.m.scaffold45.294	508	57.17	8.29	39.67	77.89	−0.255	Nuclear
LuGRAS7	L.us.o.m.scaffold45.293	319	36.11	7.09	39.48	91.29	−0.12	Nuclear
LuGRAS8	L.us.o.m.scaffold45.290	458	51.78	5.9	53.89	92.55	−0.048	Nuclear
LuGRAS9	L.us.o.m.scaffold45.289	456	51.46	5.95	52.14	92.94	−0.044	Nuclear
LuGRAS10	L.us.o.m.scaffold45.287	560	63.46	4.75	46.1	80.23	−0.243	Nuclear
LuGRAS11	L.us.o.m.scaffold14.119	472	52.52	4.79	52.97	86	−0.164	Nuclear
LuGRAS12	L.us.o.m.scaffold181.29	499	55.48	5.39	38.15	74.51	−0.475	Nuclear
LuGRAS13	L.us.o.m.scaffold218.58	759	85.22	5.79	47.94	65.3	−0.543	Nuclear
LuGRAS14	L.us.o.m.scaffold218.59	587	66.35	6.37	56.78	71.11	−0.563	Nuclear
LuGRAS15	L.us.o.m.scaffold218.61	645	73.55	6.2	49.9	65.47	−0.705	Nuclear
LuGRAS16	L.us.o.m.scaffold45.8	703	75.93	5.86	49.94	83.83	−0.276	Nuclear
LuGRAS17	L.us.o.m.scaffold227.87	409	45.29	5.81	58.42	88.68	−0.094	Nuclear
LuGRAS18	L.us.o.m.scaffold80.125	614	69.72	8.35	43.79	78.45	−0.303	Nuclear
LuGRAS19	L.us.o.m.scaffold279.14	548	61.29	5.96	45.46	77.97	−0.354	Nuclear
LuGRAS20	L.us.o.m.scaffold47.107	534	58.72	5.41	42.41	76.4	−0.322	Nuclear
LuGRAS21	L.us.o.m.scaffold47.252	458	51.84	8.95	39.84	93.41	−0.083	Nuclear
LuGRAS22	L.us.o.m.scaffold47.254	470	53.02	7.58	52.45	99.68	−0.042	Nuclear
LuGRAS23	L.us.o.m.scaffold47.258	526	59.53	5.55	42.43	87.89	−0.179	Nuclear
LuGRAS24	L.us.o.m.scaffold47.260	467	52.91	6.15	47.74	98.74	−0.073	Nuclear
LuGRAS25	L.us.o.m.scaffold47.264	470	52.9	5.71	45.86	89.23	−0.252	Nuclear
LuGRAS26	L.us.o.m.scaffold18.329	433	48.23	5.8	47.95	85.57	−0.245	Nuclear
LuGRAS27	L.us.o.m.scaffold31.240	418	46.72	7.17	46.19	93.54	−0.052	Nuclear
LuGRAS28	L.us.o.m.scaffold31.185	713	78.22	5.62	58.63	73.91	−0.322	Nuclear
LuGRAS29	L.us.o.m.scaffold90.125	523	58.09	5.88	51.19	82.68	−0.199	Nuclear
LuGRAS30	L.us.o.m.scaffold4.115	679	76.91	6.37	44.53	78.98	−0.449	Nuclear
LuGRAS31	L.us.o.m.scaffold4.412	522	57.85	5.69	49.33	81.88	−0.279	Nuclear
LuGRAS32	L.us.o.m.scaffold214.26	673	75.03	5.72	54.06	73.28	−0.529	Nuclear
LuGRAS33	L.us.o.m.scaffold17.357	570	63.06	4.95	43.95	80.61	−0.314	Nuclear
LuGRAS34	L.us.o.m.scaffold2.576	773	86.79	6.14	48.53	78.38	−0.45	Nuclear
LuGRAS35	L.us.o.m.scaffold135.44	445	48.43	5.47	48.66	93.37	−0.109	Nuclear
LuGRAS36	L.us.o.m.scaffold15.500	543	61.38	5.69	52.56	80.44	−0.328	Nuclear
LuGRAS37	L.us.o.m.scaffold15.263	738	80.71	6.1	52.67	79.46	−0.327	Nuclear
LuGRAS38	L.us.o.m.scaffold60.110	570	63.09	4.94	40.36	81.28	−0.304	Nuclear
LuGRAS39	L.us.o.m.scaffold68.132	680	73.97	6.04	46.55	70.01	−0.564	Nuclear
LuGRAS40	L.us.o.m.scaffold137.54	611	68.17	5.57	44.24	79.87	−0.355	Nuclear
LuGRAS41	L.us.o.m.scaffold100.166	476	53.22	6.1	57.53	90.02	−0.239	Nuclear
LuGRAS42	L.us.o.m.scaffold148.41	529	59.58	5.65	59.45	64.31	−0.522	Nuclear
LuGRAS43	L.us.o.m.scaffold82.222	565	61.46	5.18	54.22	78.25	−0.27	Nuclear
LuGRAS44	L.us.o.m.scaffold61.145	556	60.89	5.46	47.77	93.04	−0.115	Nuclear
LuGRAS45	L.us.o.m.scaffold27.18	542	62.09	5.56	51.63	78.58	−0.42	Nuclear
LuGRAS46	L.us.o.m.scaffold8.412	447	49.95	8.51	51.47	88.77	−0.193	Nuclear
LuGRAS47	L.us.o.m.scaffold8.414	349	39.5	8.09	33.48	79.23	−0.132	Nuclear
LuGRAS48	L.us.o.m.scaffold8.415	510	56.74	5.1	41.17	87.29	−0.276	Nuclear
LuGRAS49	L.us.o.m.scaffold8.416	528	58.57	5.44	47.63	89.38	−0.213	Nuclear
LuGRAS50	L.us.o.m.scaffold8.417	459	51.85	5.99	47.98	95.08	0	Nuclear
LuGRAS51	L.us.o.m.scaffold8.418	458	51.8	5.7	58.85	93.36	−0.078	Nuclear
LuGRAS52	L.us.o.m.scaffold8.420	592	67.63	5.26	43.83	78.34	−0.311	Nuclear
LuGRAS53	L.us.o.m.scaffold8.421	403	45.66	4.83	39.84	90.67	−0.037	Nuclear
LuGRAS54	L.us.o.m.scaffold8.427	556	63.02	4.7	43.62	84.48	−0.142	Nuclear
LuGRAS55	L.us.o.m.scaffold276.48	642	73.31	6.16	49.53	63.35	−0.715	Nuclear
LuGRAS56	L.us.o.m.scaffold276.49	762	85.62	5.79	47.43	66.57	−0.56	Nuclear
LuGRAS57	L.us.o.m.scaffold115.129	714	81.56	5.42	57.25	64.34	−0.656	Nuclear
LuGRAS58	L.us.o.m.scaffold243.14	579	64.24	5.81	48.26	76.01	−0.466	Nuclear
LuGRAS59	L.us.o.m.scaffold51.5	471	52.95	5.71	44.05	88.83	−0.263	Nuclear
LuGRAS60	L.us.o.m.scaffold51.9	491	55.5	6.27	43.38	92.51	−0.181	Nuclear
LuGRAS61	L.us.o.m.scaffold51.171	435	48.19	5.68	46.8	90.34	−0.196	Nuclear
LuGRAS62	L.us.o.m.scaffold51.237	532	58.79	5.41	42.69	77.22	−0.363	Nuclear
LuGRAS63	L.us.o.m.scaffold193.42	539	59.64	5.48	50.11	83.28	−0.239	Nuclear
LuGRAS64	L.us.o.m.scaffold29.314	453	50	5.52	43.85	89.36	−0.103	Nuclear
LuGRAS65	L.us.o.m.scaffold29.295	429	48.35	6.47	50.12	80.7	−0.324	Nuclear
LuGRAS66	L.us.o.m.scaffold133.27	593	63.69	5.03	46.62	81.05	−0.211	Nuclear
LuGRAS67	L.us.o.m.scaffold146.87	623	68.18	4.89	56.15	78.99	−0.261	Nuclear
LuGRAS68	L.us.o.m.scaffold146.102	550	61.32	5.65	36.03	84.4	−0.122	Nuclear
LuGRAS69	L.us.o.m.scaffold65.224	454	51.24	5.96	54.11	91.98	−0.291	Nuclear
LuGRAS70	L.us.o.m.scaffold69.118	472	51.89	5.38	42.26	86.14	−0.117	Nuclear
LuGRAS71	L.us.o.m.scaffold380.25	674	76.57	5.44	54.37	65.85	−0.551	Nuclear
LuGRAS72	L.us.o.m.scaffold5.93	627	68.78	6.34	56.2	68.5	−0.58	Nuclear
LuGRAS73	L.us.o.m.scaffold144.19	785	88	6.26	52.82	77.55	−0.461	Nuclear
LuGRAS74	L.us.o.m.scaffold99.186	447	48.68	5.29	49.81	92.73	−0.127	Nuclear
LuGRAS75	L.us.o.m.scaffold99.141	541	61.04	5.77	52.73	82.72	−0.328	Nuclear
LuGRAS76	L.us.o.m.scaffold76.248	737	80.83	6.05	54.42	79.96	−0.33	Nuclear
LuGRAS77	L.us.o.m.scaffold197.95	464	52.22	4.44	46.59	88.86	−0.169	Nuclear
LuGRAS78	L.us.o.m.scaffold0.697	534	59.17	6.01	44.75	97.17	−0.082	Nuclear
LuGRAS79	L.us.o.m.scaffold3.336	538	61.48	5.67	51.1	79.35	−0.419	Nuclear
LuGRAS80	L.us.o.m.scaffold13.451	681	74.23	6	51.78	72.5	−0.561	Nuclear
LuGRAS81	L.us.o.m.scaffold13.240	602	67.15	5.64	43.76	78.34	−0.382	Nuclear
LuGRAS82	L.us.o.m.scaffold238.10	481	54.69	6.45	54.39	73.18	−0.431	Nuclear
LuGRAS83	L.us.o.m.scaffold121.45	481	53.13	5.76	48.14	86.82	−0.115	Nuclear
LuGRAS84	L.us.o.m.scaffold79.112	476	53.63	5.8	56.06	91.01	−0.299	Nuclear
LuGRAS85	L.us.o.m.scaffold24.423	511	56.84	5.48	56.47	84.93	−0.155	Nuclear
LuGRAS86	L.us.o.m.scaffold147.128	423	47.36	6.13	46.7	92.25	−0.083	Nuclear
LuGRAS87	L.us.o.m.scaffold63.155	694	76.5	5.64	58.12	72.56	−0.36	Nuclear
LuGRAS88	L.us.o.m.scaffold65.63	548	60.95	5.8	37.47	83.3	−0.117	Nuclear
LuGRAS89	L.us.o.m.scaffold65.44	626	68.53	4.91	59.14	77.81	−0.281	Nuclear
LuGRAS90	L.us.o.m.scaffold39.229	473	51.98	5.44	46.71	86.98	−0.145	Nuclear
LuGRAS91	L.us.o.m.scaffold48.95	586	63.61	5.37	52.71	76.79	−0.27	Nuclear
LuGRAS92	L.us.o.m.scaffold89.34	553	60.28	5.34	46.37	94.43	−0.06	Nuclear
LuGRAS93	L.us.o.m.scaffold16.312	522	57.95	5.86	49.76	83.2	−0.198	Nuclear
LuGRAS94	L.us.o.m.scaffold122.11	702	79.44	8.1	46.1	76.27	−0.486	Nuclear
LuGRAS95	L.us.o.m.scaffold198.67	616	69.96	6.67	44.93	78.36	−0.33	Nuclear
LuGRAS96	L.us.o.m.scaffold80.60	542	60.61	5.94	44.63	79.54	−0.327	Nuclear
LuGRAS97	L.us.o.m.scaffold28.65	476	53.36	4.98	52.32	84.03	−0.174	Nuclear
LuGRAS98	L.us.o.m.scaffold46.43	496	55.28	6.85	36.2	90.44	−0.273	Nuclear
LuGRAS99	L.us.o.m.scaffold0.163	512	57.67	7.28	39.18	78.63	−0.266	Nuclear

**Figure 1.**
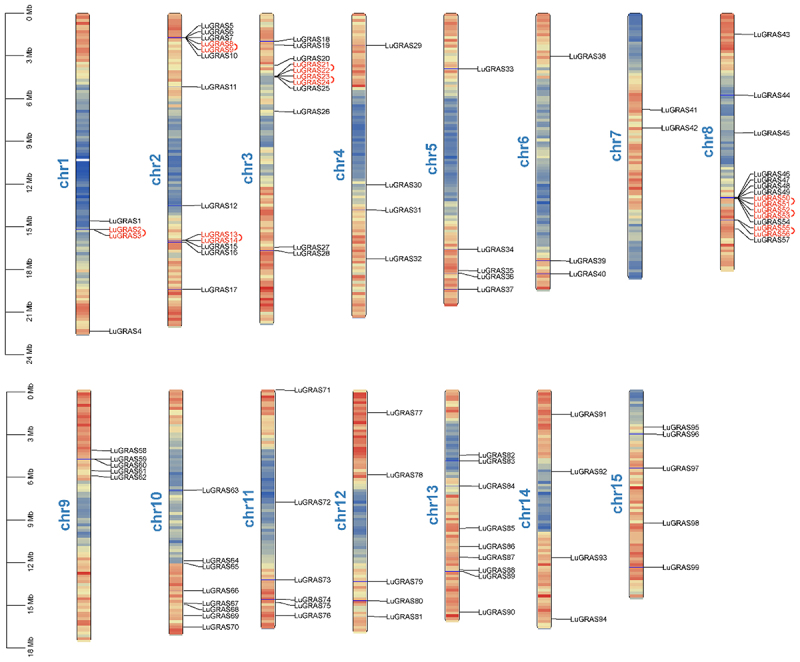
The chromosome distribution map of *LuGRAS* genes. Red genes represent pairs of tandemly duplicated genes.

The chromosomal distribution of *LuGRAS* genes was established using the flax reference genome, revealing that 99 *LuGRAS* genes are scattered unevenly across 15 chromosomes (Figure 1). Among them, the largest number of *LuGRAS* genes, 15 genes (15.15% of the total), are located on chromosome 8. The second-largest number is found on chromosome 2, which contains 13 *LuGRAS* genes (13.13% of the total). Chromosome 7 has the fewest genes, with only 2 *LuGRAS* genes (2.02% of the total).

### 3.2. Analysis of Gene Structure and Conservative Motif of LuGRAS

**Figure 2.**
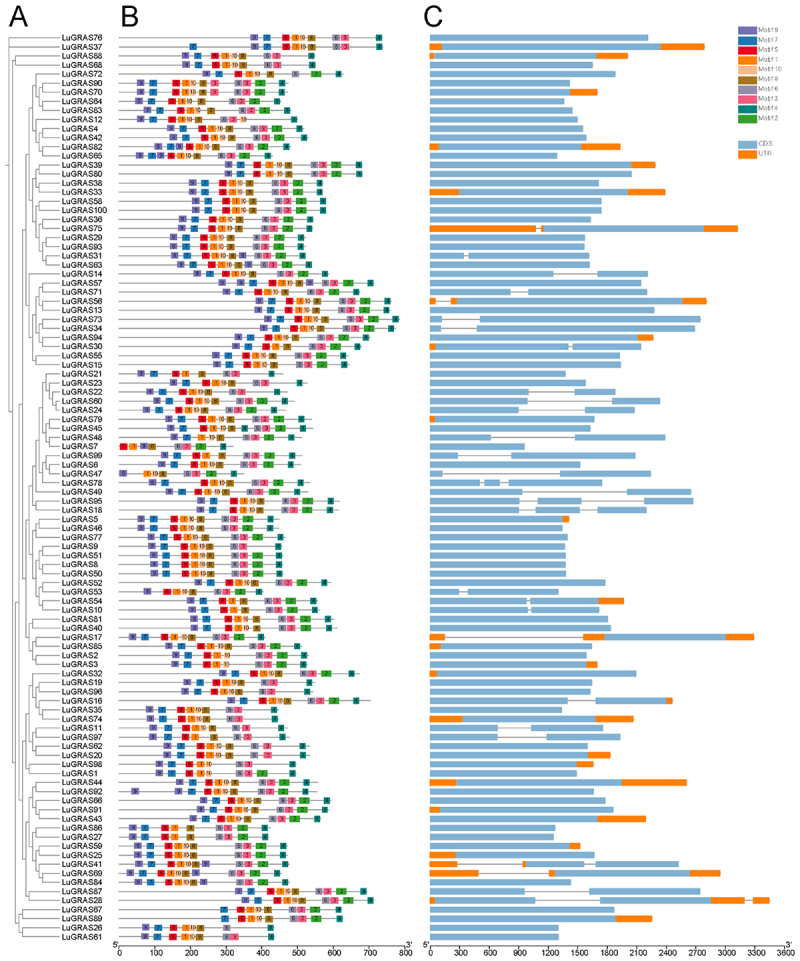
Analysis of LuGRAS gene structure and motifs. (A) phylogenetic tree of LuGRAS genes. (B) motif analysis of LuGRAS genes. The gray lines indicate the total gene length, with each conserved motif represented by a distinct color. (C) structural analysis of LuGRAS genes. The orange boxes denote UTR regions, the light blue boxes represent gene CDS regions, and the gray lines indicate introns.

To elucidate the domain architecture of *LuGRAS* family proteins, we conducted a systematic examination of evolutionarily conserved motifs across the polypeptide sequences of the entire cohort of 99 *LuGRAS* genes. A phylogenetic tree was generated based on multiple sequence alignments (Figure 2A), revealing a clustering pattern consistent with that shown in Figure 1. The results predicted 10 conserved motifs, sequentially named motif1 to motif10 (Figure 2B). Additionally, an evaluation of motifs 1–10 through Pfam revealed that motifs 1–8 are associated with Transcription Factor GRAS (Table S1). The amino acid length of the 10 motifs ranged from 15 to 29. All members contained motif1, motif4, and motif6. All members, except *LuGRAS26* and *LuGRAS72*, contained motif3, and all members, except *LuGRAS47* and *LuGRAS48*, contained motif5. Only *LuGRAS7*, *LuGRAS47*, and *LuGRAS53* lacked motif7, while the remaining members contained it. All members, except for four genes (*LuGRAS1*, *LuGRAS2*, *LuGRAS3*, and *LuGRAS98*), contained motif8. In conclusion, the motif types, quantities, and distributions of *LuGRAS* proteins within each branch were comparable. The analysis of the exon-intron structure of the *LuGRAS* genes revealed that the exon count varied from 0 to 3, while the intron count ranged from 0 to 2 (Figure 2C). Among them, *LuGRAS18*, *LuGRAS78*, and *LuGRAS95* contained the highest number of exons and introns, with 3 exons and 2 introns, respectively. Seventy-two genes (72.7% of the total) contained only one exon. Notably, most genes within the same branch had a similar number of exons, which also suggests functional similarity among these *LuGRAS* genes.

### 3.3. Phylogenetic and Collinearity Analysis of LuGRAS Gene

**Figure 3.**
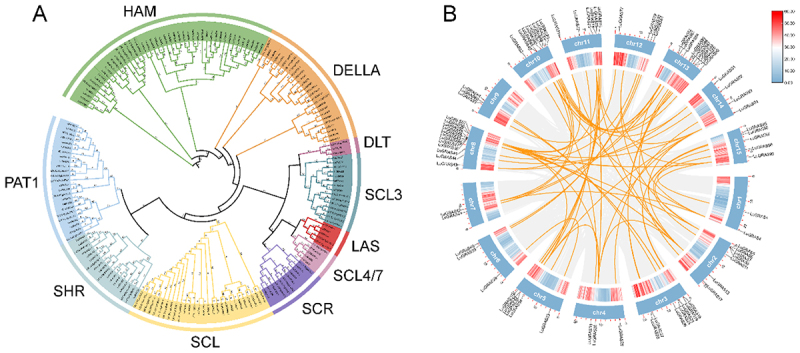
The evolutionary relationship of the GRAS gene family in flax. (A) the phylogenetic tree of LuGRAS proteins. The prefix “AT” represents arabidopsis genes, “lu” represents flax genes, and “os” represents rice genes. All GRAS genes are divided into 10 subfamilies, which are represented by different colors and letters. (B) intra-species syntenic relationships among LuGRAS members.

To resolve the evolutionary relationships within the *LuGRAS* gene family, we conducted a phylogenetic reconstruction of 182 GRAS homologs – spanning *Arabidopsis* (33), *Oryza sativa* (50), and flax (99) – using a maximum likelihood (ML)-based framework (Figure 3A). Leveraging the established *Arabidopsis* GRAS classification system, the 99 *LuGRAS* genes were phylogenetically grouped into 10 distinct clades: HAM, DELLA, DLT, SCL3, LAS, SCL4/7, SCR, SCL, SHR, and PAT1. The 99 *LuGRAS* genes were distributed across these 10 subfamilies. Among them, the HAM subfamily contained the highest number of *LuGRAS* genes (35), followed by the DELLA subfamily with 15 *LuGRAS* genes. The SCL subfamily included 11 members, whereas the DLT subfamily had the fewest *LuGRAS* genes, with only one gene. Further analysis revealed that among the three species, the GRAS family in flax was more closely related to that in *Arabidopsis*.

**Figure 4.**
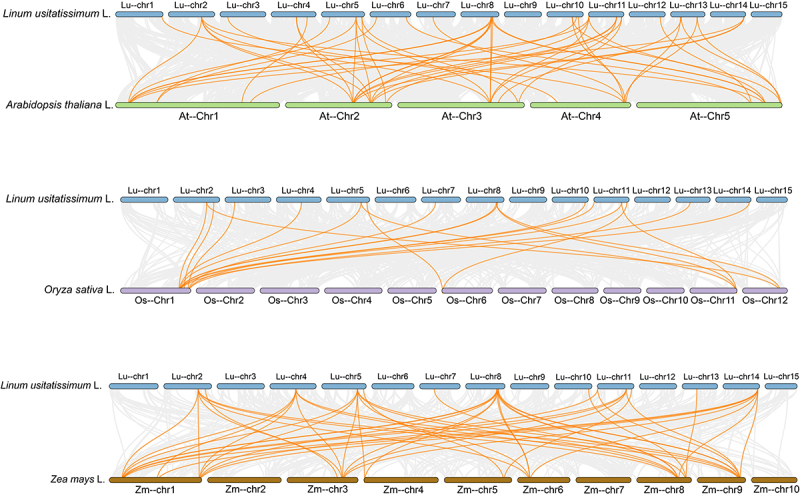
Cross-species synteny comparisons of GRAS genes between flax and three representative species: *Arabidopsis*, rice, and maize. In both panels, gray connections indicate genome-wide collinear gene pairs, while orange linkages specifically highlight syntenic pairs involving *LuGRAS* genes.

To characterize duplication patterns within the *LuGRAS* gene family, we employed the BLAST algorithm and MCScanX toolkit for systematic identification of genomic duplication events. Eight pairs of tandemly duplicated genes were identified (*LuGRAS2* and *LuGRAS3*, *LuGRAS8* and *LuGRAS9*, *LuGRAS13* and *LuGRAS14*, *LuGRAS21* and *LuGRAS22*, *LuGRAS23* and *LuGRAS24*, *LuGRAS50* and *LuGRAS51*, *LuGRAS52* and *LuGRAS53*, *LuGRAS55* and *LuGRAS56*), located on chromosomes 1, 2, 3, and 8 (Figure 3B). To elucidate genomic duplication mechanisms within the *LuGRAS* gene family, a Circos plot was generated to visualize syntenic relationships and chromosomal rearrangement patterns (Figure 3B). The results showed that there were 69 pairs of *LuGRAS* gene pairs in the flax genome, indicating significant gene family expansion in the flax GRAS family. In order to gain deeper insight into the evolutionary connections between flax and other species, we evaluated the homologous relationships between flax and three model species: *Arabidopsis*, rice, and maize (Figure 4B), to explore evolutionary differences among GRAS genes. The results showed that flax shares 51, 20, and 49 pairs of syntenic gene pairs with *Arabidopsis*, rice, and maize, respectively. Among these, five chromosomes of *Arabidopsis* exhibit synteny with all flax chromosomes except chromosomes 9 and 15; the syntenic GRAS gene pairs in rice are located on flax chromosomes 2–5, 7–8, 10–11, 13, and 14; and maize GRAS syntenic gene pairs are located on flax chromosomes 2, 4–5, 7–8, 10–11, 13, and 14. In summary, the GRAS genes of flax and the three representative species exhibit mostly conserved synteny across chromosome regions, although there are also differences in the gene pairs, highlighting the important role of *LuGRAS* gene family evolution.

### 3.4. Analysis of Cis-Acting Elements and mirna Prediction

**Figure 5.**
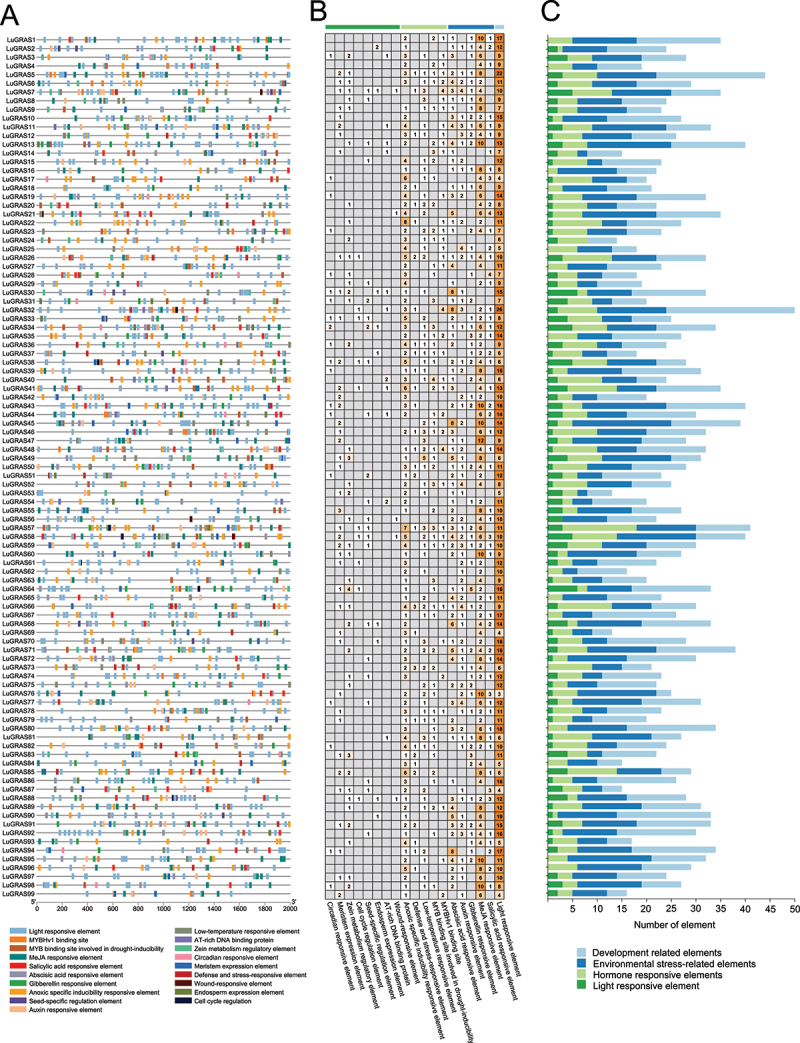
Analyzes cis-elements in *LuGRAS* promoters. (A) subfamily distribution. (B, C) element quantification by category: light blue (developmental), dark blue (stress), light green (hormone), dark green (light response).

To investigate the functional mechanisms of the *LuGRAS* gene family in abiotic stress adaptation, we conducted a comprehensive examination of promoter regions spanning 2000 base pairs upstream of each *LuGRAS* locus through bioinformatic scrutiny (Figure 5A). Following the exclusion of ubiquitous core promoter elements (TATA-box and CAAT-box), 2,650 cis-regulatory motifs were detected and classified into four functional categories: developmental regulation, stress adaptation, phytohormone signaling, and light response (Figure 5B-C; Table S2). Notably, light-responsive motifs dominated the profile (1,065 elements, 40.19%), featuring conserved sequences such as Box 4, G-box, GT1-motif, and TCT-motif. Phytohormone-associated elements ranked second (881 motifs, 33.25%), predominated by TGACG-motif and CGTCA-motif associated with methyl jasmonate (MeJA) response, along with ABRE (abscisic acid), TGA-element/AuxRR-core (auxin), GARE-motif/P-box (gibberellin), and TCA-element (salicylic acid). Stress adaptation motifs (509 elements, 19.21%) comprised regulatory sequences linked to anaerobic induction (ARE, GC-motif), low-temperature response (LTR), drought-inducible MYB binding sites (MBS), MYBHv1 recognition sites (CCAAT-box), and defense/stress signaling (TC-rich repeats). Developmental regulation elements (195 motifs, 7.36%) included meristem-specific CAT-box, zein metabolism controllers (O2-site), endosperm-active motifs (GCN4-motif, AACA-motif), circadian rhythm regulators, AT-rich DNA binding domains, cell cycle modulators (MSA-like), seed-specific RY-element, and wound-inducible WUN-motif.

The miRNA prediction analysis revealed that among the 99 members of the *LuGRAS* gene family, only 31 (31.31%) were predicted to harbor 62 miRNA targets (Table S3). The genes *LuGRAS56* and *LuGRAS18* exhibited the highest number of predicted miRNA targets (10 each), including lus-miR167b/c/d/e/f/g/h/i, lus-miR530a/b, and lus-miR166a/c/d/e/f/g/h/i/j/k. In contrast, nine genes (*LuGRAS1*, *7*, *11*, *26*, *35*, *49*, *68*, *73*, and *74*) contained the fewest miRNA targets, with only one predicted site per gene. Notably, individual *LuGRAS* genes could be targeted by distinct miRNAs. For example, *LuGRAS10* was simultaneously targeted by lus-miR168 and lus-miR159. Conversely, identical miRNAs were observed to target multiple *LuGRAS* genes. Specifically, lus-miR394 targeted four genes (*LuGRAS25*, *28*, *59*, and *87*), while lus-miR395 also regulated four genes (*LuGRAS1*, *35*, *54*, and *74*). Collectively, lus-miR395 emerged as the predominant miRNA targeting the *LuGRAS* gene family.

### 3.5. Analysis of LuGRAS Gene Expression Patterns Based on RNA-Seq Data

**Figure 6.**
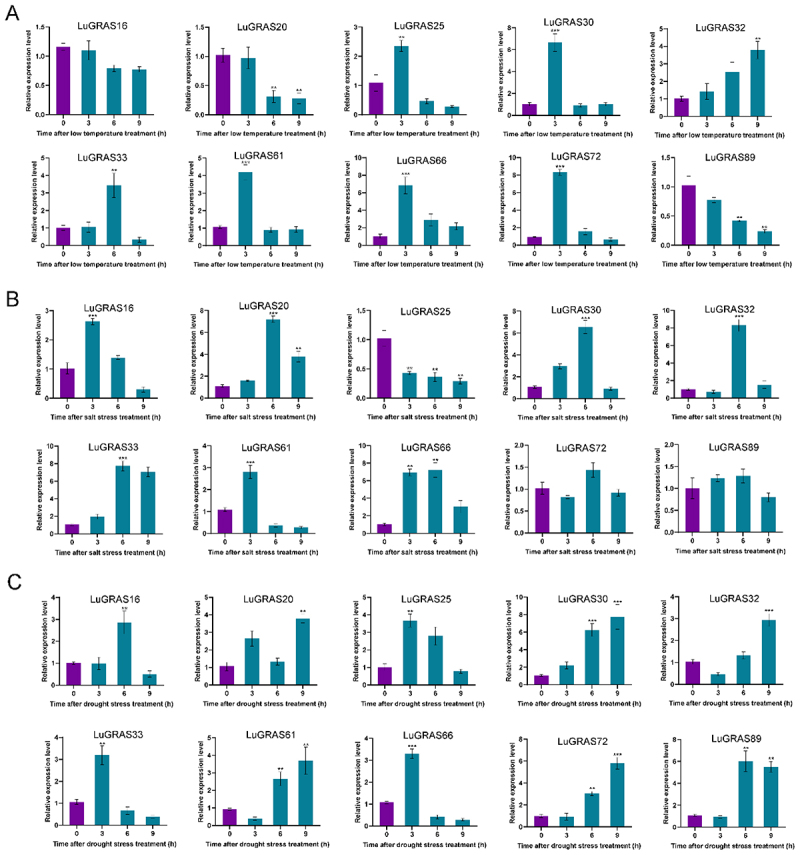
Transcriptional dynamics of *LuGRAS* family members under abiotic stress. (A) cold stress response, (B) salt stress response, and (C) drought stress response. Violet histograms denote untreated controls, while cerulean histograms represent stress-exposed samples. Differential expression between groups was analyzed via Student’s t-test, with asterisks marking significance thresholds (**p < .01; ***p < .001).

To explore the functional contributions of *LuGRAS* genes under abiotic stress, we assessed their transcriptional dynamics during salt and thermal stress using open-access RNA-seq datasets (Figure 6A). The *LuGRAS* gene family exhibited no significant changes under heat stress, with only *LuGRAS43*, *LuGRAS88*, and *LuGRAS92* showing marked upregulation. In contrast, most *LuGRAS* genes displayed significant alterations in both leaf and root tissues under salt stress. Compared to the control, four genes (*LuGRAS29*, *31*, *58*, and *98*) were upregulated in root tissues under salt stress, with *LuGRAS58* showing the most pronounced differential expression. In leaf tissues, 14 genes (*LuGRAS15*, *31*, *33*, *35*, *36*, *38*, *54*, *58*, *63*, *73*, *80*, *88*, *94*, and *98*) were significantly upregulated under salt stress, with *LuGRAS15* exhibiting the highest differential expression.

To delineate the functional diversity of the *LuGRAS* gene family in plant morphogenesis and developmental regulation, we performed a systematic transcriptomic profiling of all annotated *LuGRAS* homologs across 10 distinct tissue types utilizing publicly available RNA sequencing datasets (Figure 6B). The expression profiles of *LuGRAS* genes varied among tissues. Most *LuGRAS* genes were highly expressed in leaf and root tissues. Two members (*LuGRAS58* and *LuGRAS75*) showed high expression in stem tissues, while two genes (*LuGRAS3* and *LuGRAS90*) were highly expressed in ovary tissues. Eight members (*LuGRAS2*, *LuGRAS7*, *LuGRAS10*, *LuGRAS26*, *LuGRAS40*, *LuGRAS50*, *LuGRAS51*, and *LuGRAS99*) exhibited high expression in anther tissues. *LuGRAS2* and *LuGRAS9* were specifically highly expressed in stamen and mature embryo tissues, respectively. Three members (*LuGRAS5*, *LuGRAS18*, and *LuGRAS23*) were strongly expressed in pistil tissues. Additionally, *LuGRAS10* and *LuGRAS87* were highly expressed in fruit tissues, while *LuGRAS22* and *LuGRAS23* showed elevated expression in seed tissues. Collectively, the predominant expression of most *LuGRAS* genes in leaf and root tissues suggests that flax leaves and roots may serve as primary sites for regulating ROS homeostasis and act as the first line of antioxidant defense in plants, with *LuGRAS* genes playing critical regulatory roles in these processes.

### 3.6. Expression Level of LuGRAS Gene in Flax Under Abiotic Stress

To delineate functional divergence within the *LuGRAS* gene family, 10 representative homologs spanning 10 subfamilies exhibiting high evolutionary conservation with *Arabidopsis* GRAS orthologs were prioritized. QRT-PCR assays demonstrated temporally variable transcriptional responses of these selected genes under abiotic stress challenges. Under cold stress (Figure 6A), all nine tested *LuGRAS* genes exhibited significant temporal responses. Five genes (*LuGRAS25*, *LuGRAS30*, *LuGRAS61*, *LuGRAS66*, and *LuGRAS72*) peaked at 3 h, showing 2.2-, 6.3-, 4.1-, 6.7-, and 8.1-fold increases, respectively, followed by rapid declines. *LuGRAS33* and *LuGRAS32* reached maxima at 6 h (3.2-fold) and 9 h (3.6-fold), while *LuGRAS20* and *LuGRAS89* progressively decreased, suggesting negative regulatory roles. Under salt stress (Figure 6B), *LuGRAS16* and *LuGRAS61* peaked at 3 h (2.5- and 2.7-fold increases), while five genes (*LuGRAS25*, *LuGRAS30*, *LuGRAS61*, *LuGRAS66*, and *LuGRAS72*) reached peak expression at 6 h (6.9-, 6.2-, 7.9-, 7.3-, and 7.1-fold increases) before declining. These results demonstrate stress-specific regulatory dynamics of *LuGRAS* genes, highlighting their roles in abiotic stress adaptation.

As the duration of drought stress increased, the expression levels of all *LuGRAS* genes showed significant changes (Figure 6C). Three genes (*LuGRAS25*, *LuGRAS33*, and *LuGRAS66*) reached their peak expression at 3 hours of drought stress, increasing approximately 3.6-fold, 3.1-fold, and 3.2-fold compared to the control group, respectively. *LuGRAS16* and *LuGRAS89* reached their highest expression levels at 6 hours of drought stress before rapidly declining, with increases of approximately 2.8-fold and 5.8-fold compared to the control, respectively. Five genes (*LuGRAS20*, *LuGRAS30*, *LuGRAS32*, *LuGRAS61*, and *LuGRAS72*) exhibited peak expression at 9 hours of drought stress, with expression levels increasing by approximately 3.7-fold, 7.2-fold, 2.8-fold, 3.4-fold, and 5.7-fold compared to the control, respectively.

### 3.7. Constructing Transgenic Plants with LuGRAS30 Gene and Its Role in Drought Stress Response

**Figure 7.**
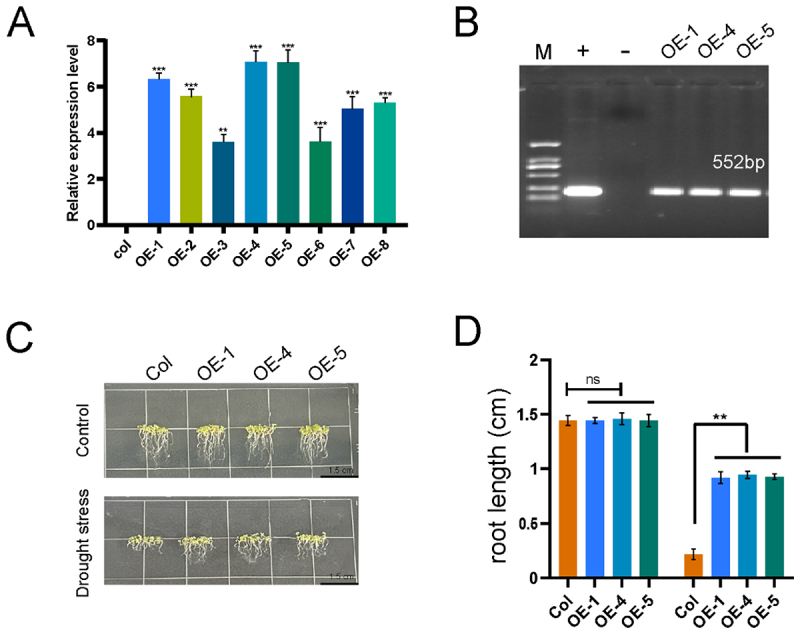
Functional characterization of *LuGRAS30*-overexpressing *arabidopsis* in drought adaptation. (A) PCR validation of *LuGRAS30* gene expression in 35S:*LuGRAS30* overexpression plants (OE-1 to OE-8). (B) amplification of the bar selection marker in representative lines (OE-1, OE-4, OE-5) validated transgene integration. Lane annotations: *M* – DL2000 DNA ladder; “+” – recombinant pCAMBIA3301-LuGRAS30 plasmid; “”- – empty vector control. (C, D) comparative root elongation analysis of *LuGRAS30*-transgenic *arabidopsis* under drought stress (scale bar: 1.5 cm). Statistically significant differences between transgenic and wild-type plants were determined by Student’s t-test (*p < .05; **p < .01).

In this study, we observed that the *LuGRAS30* gene exhibited strong stress resistance. To investigate its function, the recombinant plasmid pCAMBIA3301-LuGRAS30 was constructed and transformed into wild-type *Arabidopsis* Col plants. Eight transgenic lines overexpressing *LuGRAS30* were identified. Molecular analysis of T3-generation transgenic plants revealed three homozygous lines (OE-1, OE-4, and OE-5) with the highest expression levels (Figure 7(A,B)). Root growth assays showed that both Col and *LuGRAS30*-overexpressing transgenic *Arabidopsis* grew normally on standard 1/2 MS medium, with no significant differences in primary root length. However, under mannitol treatment (200 mM), transgenic *Arabidopsis* overexpressing *LuGRAS30* displayed enhanced drought tolerance compared to wild-type plants (Figure 7(C,D)).

### 3.8. Subcellular Localization of LuGRAS30 Gene

**Figure 8.**
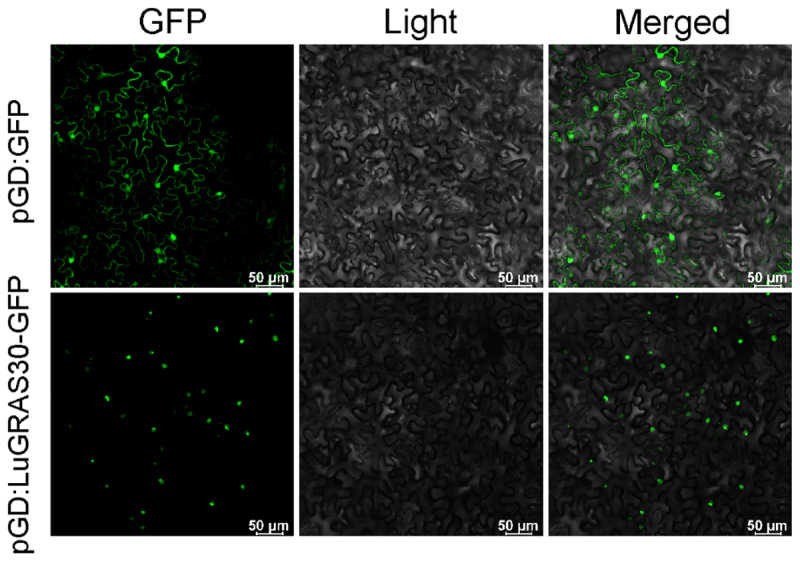
Analysis of the subcellular localization of the *LuGRAS30* protein in tobacco cells. Scale bar = 50µm.

The intracellular localization of *LuGRAS30* was determined through transient transformation assays in young *Nicotiana benthamiana* leaves using *Agrobacterium tumefaciens* GV3101 harboring either the GFP-tagged fusion construct pCAMBIA1301-LuGRAS30-GFP or the empty vector control (pCAMBIA1301-GFP). The GFP signal in the empty vector control was distributed throughout the cell, while LuGRAS30-GFP localized specifically to the nucleus (Figure 8), consistent with the prediction in Table 1.

### 3.9. Overexpression of LuGRAS30 Gene Reduces ROS Accumulation

**Figure 9.**
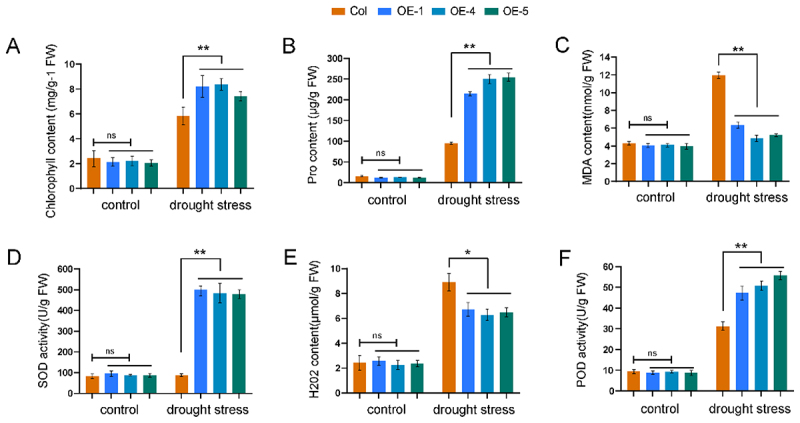
Physiological profiling of *LuGRAS30*-overexpressing *arabidopsis* lines. (A) chlorophyll content, (B) proline (Pro) levels, (C) malondialdehyde (MDA) accumulation, (D) superoxide dismutase (SOD) activity, (E) hydrogen peroxide (H₂O₂) concentration, and (F) peroxidase (POD) activity in *LuGRAS30*-overexpressing lines versus wild-type controls. Data represent means ± SD from three biological replicates. Statistically significant differences (*p < .05, **p < .01) were determined via Student’s t-test.

To investigate the physiological and biochemical effects of the *LuGRAS30* gene in overexpression plants, chlorophyll content, proline (Pro) content, and malondialdehyde (MDA) content were measured in Col and OE lines (Figure 9A-C). Under drought stress, the OE lines (OE-1, OE-4, and OE-5) exhibited increased chlorophyll content, significantly reduced MDA levels, and significantly elevated Pro content compared to Col plants. To explore the effect of *LuGRAS30* on reactive oxygen species (ROS) accumulation, hydrogen peroxide (H₂O₂) content, superoxide dismutase (SOD) activity, and peroxidase (POD) activity were analyzed (Fig 9D-F). The results showed no significant differences in SOD activity, H₂O₂ content, and POD activity between Col and OE lines under normal conditions. However, under drought stress, OE lines displayed significantly higher SOD and POD activities alongside markedly reduced H₂O₂ levels. These findings suggest that *LuGRAS30* enhances plant tolerance to abiotic stresses by upregulating peroxidase and antioxidant enzyme activities in leaves, thereby mitigating ROS accumulation.

### 3.10. Expression Pattern Analysis of Key Genes in Drought Stress Pathway in oe-LuGRAS30 Strain

**Figure 10.**
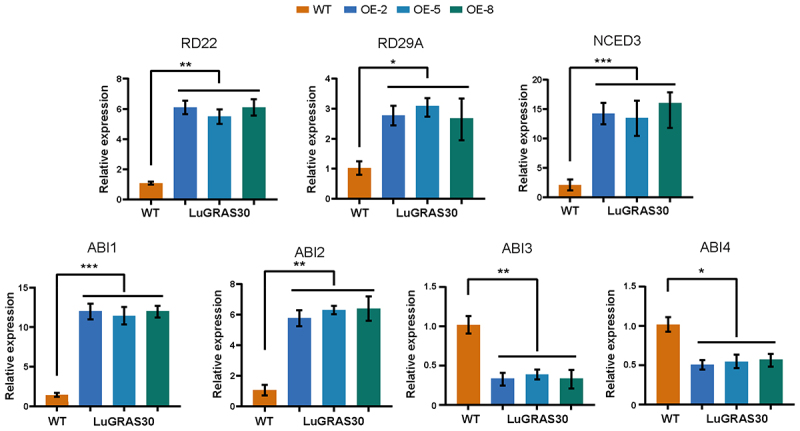
Transcriptional profiling of drought-responsive genes in *LuGRAS30*-overexpressing (OE-*LuGRAS30*) and wild-type (Col) *Arabidopsis*. The analyzed genes included *RD22*, *RD29A*, *NCED3*, *ABI1*, *ABI2*, *ABI3*, and *ABI4*. Data are expressed as mean ± standard deviation (SD) from three independent biological replicates. Statistically significant differences between groups are denoted by asterisks: *p < .05, **p < .01 (Student’s t-test).

To delineate molecular adaptations to drought stress, we quantified the transcriptional activity of seven drought-responsive marker genes (*RD22*, *RD29A*, *NCED3*, *ABI1*, *ABI2*, *ABI3*, and *ABI4*) in *LuGRAS30*-overexpressing transgenic lines (OE-*LuGRAS30*), revealing their regulatory interplay under water-deficit conditions (Figure 10). The results showed that the expression of *ABI3* and *ABI4* was suppressed under drought stress, with reduced transcript levels. In contrast, five key genes (*RD22*, *RD29A*, *NCED3*, *ABI1*, and *ABI2*) exhibited significant upregulation, with expression levels higher than those in wild-type plants. These findings suggest that *LuGRAS30* enhances drought resistance in transgenic plants through the ABA biosynthesis pathway or other regulatory mechanisms.

## 4. Discussion

GRAS transcription factors (TFs) play critical roles in modulating diverse plant processes, including growth regulation, developmental programming, and environmental stress adaptation.^35^ Despite extensive characterization of GRAS genes across angiosperms, their functional landscape in flax remains largely unexplored. Notably, no systematic genome-wide analysis of GRAS genes in flax has been documented. Here, through comprehensive genomic screening, we annotated 99 putative GRAS homologs (*LuGRAS1*–*LuGRAS99*) within the Longya10 flax genome (Table 1). Our analysis revealed that the number of *LuGRAS* genes in flax (99) was significantly lower than that in apple (127),^36^ while being comparable to poplar (106)^37^ and maize (86).^38^ Notably, the *LuGRAS* gene family in flax was substantially larger than that in *Arabidopsis* (33),^39^ rice (57),^40^ *Brassica rapa* (48),^41^ tobacco (53),^42^ *Prunus mume* (46),^43^ and castor (48).^44^ Moreover, the 99 *LuGRAS* genes were unevenly distributed across 15 chromosomes (Figure 1).

According to the results of the conserved motif analysis, most *LuGRAS* proteins exhibited a similar motif order (Figure 2B). This pattern consisted of the sequence of motif9, motif7, motif5, motif1, motif6, motif3, and motif4, among which motif1–8 were associated with the function of the Transcription factor GRAS. This finding was consistent with the study in *Phoebe bournei*.^45^ Analysis of gene architecture demonstrated that *LuGRAS* homologs exhibited exon counts ranging from 0 to 3 and intron numbers varying between 0 and 2 (Figure 2C). While the majority displayed conserved structural configurations, observed divergence in GRAS gene organization may reflect evolutionary dynamics driven by exon/intron indels or splicing variations.^46^ We found that each evolutionary branch of the *LuGRAS* genes exhibited a conserved exon-intron motif, which suggested that *LuGRAS* genes played similar roles in various abiotic stress-related responses. This finding was consistent with similar observations in cassava.^47^

The evolution of GRAS genes was significantly influenced by gene duplication events, which may have led to the acquisition of novel functions in duplicated genes, ultimately strengthening plant responses to environmental stresses.^48^ In this study, the phylogenetic analysis results indicated that the *LuGRAS* genes were divided into 10 subfamilies (Figure 3A), which was generally consistent with previous studies.^41^ Additionally, 69 pairs of *LuGRAS* genes were found in the flax genome (Figure 3B). These findings suggest that the expansion of the *LuGRAS* gene family may have been predominantly driven by large-scale genomic duplication events.^49^ Comparative synteny analysis, a methodology widely used to reconstruct evolutionary trajectories of gene families,^50^ identified 51, 20, and 49 orthologous gene pairs between flax GRAS homologs and their counterparts in *Arabidopsis*, rice, and maize, respectively (Figure 4). This finding suggests that these homologous gene pairs may have originated from a common ancestor predating species divergence.^51^ It was demonstrated that the *LuGRAS* genes are highly conserved in flax, and it was speculated that the expansion of *LuGRAS* genes was mainly caused by gene fragment duplication.

Cis-regulatory elements within promoter regions orchestrate transcriptional regulation, primarily through the sequence-specific binding of transcription factors to their cognate motifs.^52^ Bioinformatic interrogation of *LuGRAS* promoter sequences in this study revealed a dominant enrichment of cis-elements associated with phytohormonal signaling pathways (methyl jasmonate and abscisic acid) and abiotic stress adaptation mechanisms, including hypoxia response, low-temperature acclimation, and drought tolerance (Figure 5). Among the elements involved in stress responses, LTR was associated with plant adaptation to low temperatures, ARE elements were linked to anaerobic induction, and TC-rich repeats were correlated with drought resistance.^53^ The promoters of *LuGRAS* genes contain abscisic acid-responsive elements (ABRE), methyl jasmonate (MeJA)-responsive elements (TGACG-motif and CGTCA-motif), and MYB binding sites (MBS) involved in drought induction. This indicates high conservation of these cis-elements and suggests their critical roles in regulating stress-related hormonal signaling or biotic/abiotic stress responses.^54^

MicroRNAs function as pivotal modulators in synchronizing plant developmental processes and mediating biotic/abiotic stress adaptation.^55^ Computational prediction identified lus-miR395 as the predominant miRNA targeting *LuGRAS* family members (Table S3). Studies have shown that in *Arabidopsis*, miR395, through a multi-target synergistic mechanism, became a core component of the plant sulfate assimilation regulatory network. The expression pattern of miR395 under sulfur deficiency stress and its regulation of the target gene APS1 played an important role in oxidative stress.^56^ The regulatory mechanism of the GRAS gene and the miRNA159-GRAS module requires further study.

**Figure 11.**
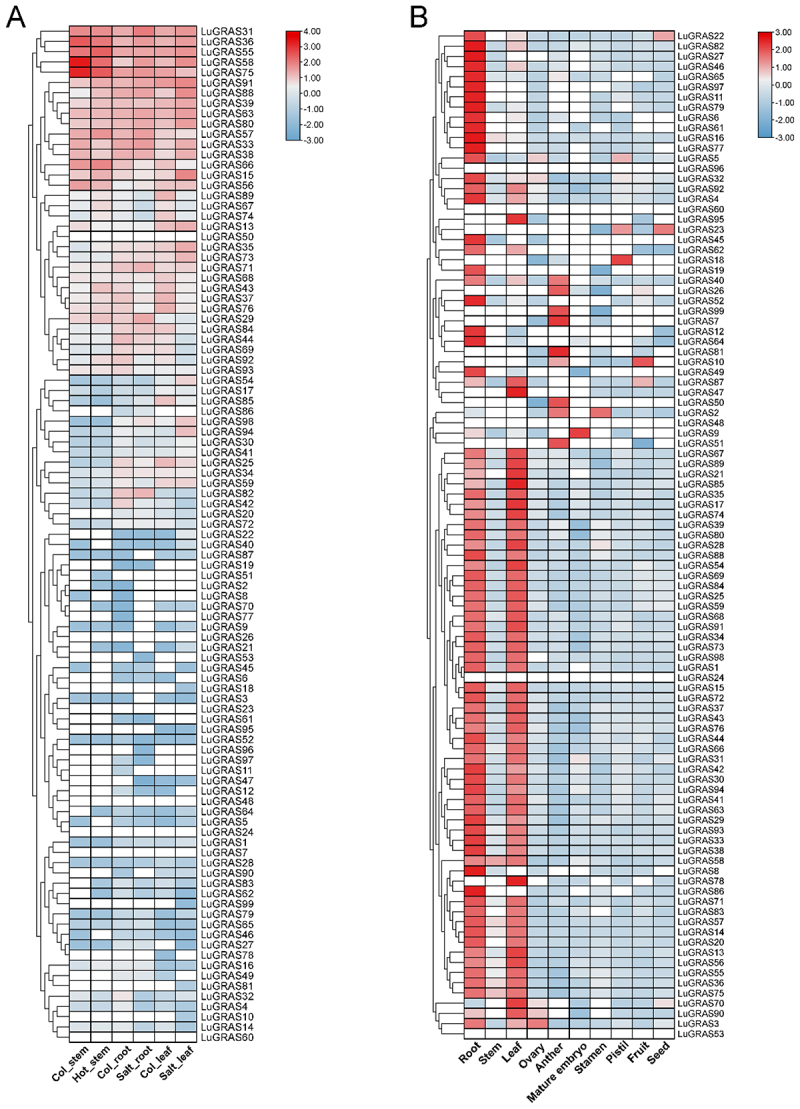
Transcriptional profiling of the *LuGRAS* gene family. (A) expression dynamics of *LuGRAS* homologs under NaCl and thermal stress treatments. (B) tissue-specific transcriptional activity of *LuGRAS* genes across distinct organs of flax. Expression levels, quantified as log2-transformed FPKM values, are visualized through a color gradient from elevated (red) to diminished (blue) expression intensities.

RNA-seq data analysis revealed tissue-specific variations in *LuGRAS* gene expression, with the majority of *LuGRAS* genes exhibiting high expression levels in leaves and roots (Figure 11). Studies had shown that in *Arabidopsis*, *SCR* and *SHR* regulated the radial differentiation of cortical and stele cells, playing a dominant role in root patterning.^9^ The GRAS protein *PAT1* in tomato orchestrates light-mediated leaf morphogenesis by modulating interactions with phytochrome-associated transcription factors (PIFs).^57^ These findings underscore the functional diversification of GRAS genes in developmental regulation, with select members demonstrating tissue-specific roles during organogenesis.

The GRAS gene family, which is widely distributed across diverse plant taxa, is known for its multifunctional roles in plant biology. In flax, beyond their established involvement in growth and development, *LuGRAS* genes contribute significantly to the plant’s adaptation to abiotic stress. Transcriptional profiling under salt stress conditions revealed marked upregulation of four *LuGRAS* homologs (*LuGRAS29*, *31*, *58*, and *98*) in root tissues compared to unstressed controls, with *LuGRAS58* exhibiting the most pronounced differential expression. In the leaf tissue of flax, 14 genes (*LuGRAS15*, *31*, *33*, *35*, *36*, *38*, *54*, *58*, *63*, *73*, *80*, *88*, *94*, and *98*) showed significant increases under salt stress, with the most significant differential expression observed for the *LuGRAS15* gene (Figure 11). We further validated this through qRT-PCR. Under cold stress, the expression of five genes (*LuGRAS25*, *LuGRAS30*, *LuGRAS61*, *LuGRAS66*, and *LuGRAS72*) peaked at 3 hours of low-temperature stress and then rapidly decreased (Figure 6A). Under salt stress, the expression of five genes (*LuGRAS25*, *LuGRAS30*, *LuGRAS61*, *LuGRAS66*, and *LuGRAS72*) peaked at 6 hours of salt stress and then quickly decreased (Figure 6B). Under drought stress, the expression of five genes (*LuGRAS20*, *LuGRAS30*, *LuGRAS32*, *LuGRAS61*, and *LuGRAS72*) peaked at 9 hours of drought stress (Figure 6C). Earlier studies demonstrated notable variations in the expression levels of 12 genes in *Dendrobium chrysotoxum* when subjected to high temperature, drought, and salt stress. Among these, *DchGRAS13* and *DchGRAS15* exhibited the highest sensitivity to environmental stressors.^58^ Transcriptional profiling of Phoebe bournei under abiotic stress conditions revealed significant upregulation of four *PbGRAS* homologs (*PbGRAS7*, *PbGRAS10*, *PbGRAS14*, and *PbGRAS16*) during water deficit, salinity, and thermal stress treatments.^45^ These results collectively highlight the critical involvement of GRAS transcription factors in mediating stress adaptation mechanisms in flax

Ectopic expression of *LuGRAS30* in transgenic plants conferred enhanced drought resilience by modulating stress-responsive pathways, as evidenced by phenotypic and molecular analyses under water-deficit conditions (Figure 7). This aligns with prior findings where heterologous expression of *PeSCL7* from *Populus euphratica* in *Arabidopsis* markedly elevated tolerance to both osmotic and ionic stresses, underscoring the conserved role of GRAS transcription factors in abiotic stress adaptation across divergent plant taxa.^59^ *SIGRAS4* enhanced the cold tolerance of tomato through a dual mechanism: it directly regulated the expression of multiple cold acclimation-related genes and induced the expression of the key regulatory factor *SICBF*, thereby synergistically improving the plant’s cold resistance.^60^ ROS were pivotal in preserving cellular equilibrium and facilitated intracellular signaling across biological systems. Under abiotic stress, plants exhibited elevated ROS accumulation – encompassing enzymes such as peroxidase and metabolites like hydrogen peroxide (H₂O₂) – which resulted in oxidative stress and subsequent cellular injury.^61^ Under drought stress, the OE lines (OE-1, OE-4, and OE-5) exhibited increased chlorophyll content, significantly reduced malondialdehyde (MDA) levels, and elevated proline (Pro) content compared to Col plants. Additionally, these lines demonstrated markedly higher superoxide dismutase (SOD) and peroxidase (POD) activities, along with significantly lower hydrogen peroxide (H₂O₂) accumulation (Figure 9). These findings suggest that the heterologous expression of *LuGRAS30* contributes to alleviating abiotic stress by facilitating the removal of ROS. Furthermore, under drought stress, the expression levels of *ABI3* and *ABI4* were suppressed, whereas five key genes (*RD22*, *RD29A*, *NCED3*, *ABI1*, and *ABI2*) were significantly upregulated, with expression levels exceeding those in wild-type plants (Figure 10). Reports indicated that *RD22* was a key marker of gene expression triggered by ABA, whereas *RD29A* was activated under drought and salt stress and exhibited responsiveness to ABA treatment.^62^ The enzyme *NCED3* acted as a key regulatory bottleneck in the ABA biosynthetic cascade, governing the transcriptional activation of drought-inducible genes.^63^ Together, these results demonstrate that *LuGRAS30* overexpression augments drought resilience in plants by fine-tuning ABA-mediated signaling networks, dynamically regulating stress-responsive gene expression, and orchestrating transcriptional remodeling coupled with enhanced antioxidant defense systems under abiotic stress.

## 5. Conclusions

This study represents the first systematic investigation of the GRAS gene family in flax. Genome-wide screening identified 99 GRAS homologs, which were phylogenetically classified into 10 distinct clades. Synteny analysis revealed extensive duplication events, including 8 tandemly duplicated loci and 69 segmentally duplicated gene pairs. Computational miRNA targeting predicted lus-miR395 as the predominant regulatory miRNA interacting with *LuGRAS* members. Promoter profiling demonstrated that most *LuGRAS* genes harbor cis-regulatory motifs linked to phytohormone signaling (e.g., MeJA, ABA) and abiotic stress responses. Transcriptomic and qRT-PCR validation further highlighted their functional divergence in organ-specific development (roots, leaves), hormonal regulation, and adaptation to cold, drought, and salt stresses. Functional characterization of *LuGRAS30* in transgenic *Arabidopsis* demonstrated its role in enhancing drought tolerance by mitigating ROS accumulation. Collectively, this work establishes a foundational framework for understanding the evolutionary dynamics and stress-responsive mechanisms of GRAS genes in flax, providing molecular insights for crop improvement strategies.

## Supplementary Material

TableS4.xlsx

TableS2.xlsx

TableS3.xlsx

TableS1.xlsx

## Data Availability

All data are reported in the article and Supplementary Materials.
